# Personalized Lifespan Approach to Autism Assessment: A Clinical and System Framework for Developmentally Informed Assessment Across the Life‐Course

**DOI:** 10.1002/aur.70294

**Published:** 2026-06-19

**Authors:** Melanie Penner, Rebecca McNally Keehn

**Affiliations:** ^1^ Holland Bloorview Kids Rehabilitation Hospital Toronto Ontario Canada; ^2^ Department of Paediatrics University of Toronto Toronto Ontario Canada; ^3^ Department of Pediatrics Indiana University School of Medicine Indianapolis Indiana USA

**Keywords:** assessment, autism, diagnosis, health services, lifespan

## Abstract

The increasing prevalence of autism has led to considerable system challenges as specialist‐driven approaches have struggled to keep pace. Recent research has challenged status quo models by demonstrating that community‐based pediatric clinicians can accurately diagnose autism in young children. This research provides an opportunity to further expand capacity of community systems to conduct developmentally relevant, context‐specific assessments of autistic people over their lifespan. In this Commentary, we propose a *personalized lifespan approach to autism assessment* as a precision care and system organization framework that considers the ongoing and evolving needs of autistic people and aligns the necessary expertise and resources to provide focused assessment when it is most informative. This framework consists of three key principles: (1) assessment is not a one‐time, one‐size‐fits‐all event; instead, assessment continues across the lifespan; (2) assessments should generate information that is relevant to the individual's current needs and life stage; and (3) wherever possible, community‐based expertise should be engaged to promote the right assessment at the right time in the right place. Adopting this framework can reduce system access challenges, while also addressing the ongoing assessment needs of an autistic person across their life course.

## Summary

Historical models of autism care have not kept up with the demand for autism diagnostic evaluations, leading to lengthy wait times. Recent research has shown that community‐based pediatric clinicians can diagnose autism with high accuracy. In this paper, we suggest expanding these findings into a *personalized lifespan approach to autism assessment* that takes a broader view of assessment as occurring across many points over a person's lifetime and in a person's community whenever possible. This model can help to ensure that autistic people have ongoing access to learn more about themselves, and that they can get these answers when they need them and closer to home.

Autism prevalence has increased by 400% in the past 20 years (Shaw et al. 2025), but the systems in place to support diagnosis have not kept pace (Ning et al. 2019). The traditional diagnostic pathway—starting with identification of possible autism by a community‐based pediatric clinician (e.g., pediatrician, primary care clinician, community‐based consultant) followed by referral to a specialist for a diagnostic evaluation (Hyman et al. 2020)—has led to substantial delays in care. The number of children requiring autism evaluation far exceeds available specialists (Bridgemohan et al. 2018; Weitzman et al. 2022), with greater access challenges for those from minoritized backgrounds or rural and under‐resourced regions (Murphy and Ruble 2012; Drahota et al. 2020; Smith et al. 2020; Aylward et al. 2021). The majority of US diagnostic centers have waitlists that exceed nine months (Cognoa Editorial Team 2023); data from Canada (BC Provincial Health Services Authority 2022) and the UK (NHS England 2024) show similar access challenges. Hundreds of thousands of young children per year wait for a diagnostic evaluation, unable to access services that confer developmental advantage (Aylward et al. 2021; Zeleke et al. 2019; Wiggins et al. 2020), relieve family stress (Bejarano‐Martín et al. 2020), and reduce long‐term costs (Petruccelli et al. 2022; Patten et al. 2013; Pickard and Ingersoll 2016; Porterfield and McBride 2007; Vu et al. 2023). Innovative solutions and system changes that challenge our historical approach to diagnostic evaluation are critically needed (Zwaigenbaum and Warren 2021). While categorical autism diagnosis (i.e., determining autism presence or absence) is a critical step in the assessment process that provides families with answers and access to autism‐focused interventions, autistic individuals require ongoing developmentally‐focused assessment to guide support planning across the lifespan.

In this Commentary, we propose a *personalized lifespan approach to autism assessment* as a precision care and system organization framework that considers the ongoing and evolving needs of autistic people and aligns the necessary expertise and resources to provide focused assessment when it is most informative. This framework consists of three key principles guiding *when* and *where* assessment occurs: (1) assessment is not a one‐time, one‐size‐fits‐all event; instead, assessment continues across the lifespan; (2) assessments should generate information that is relevant to the individual's current needs and life stage; (3) wherever possible, community‐based expertise should be engaged to promote the right assessment at the right time.

## 1 Historical Approaches to Autism Assessment

Early models of autism diagnostic assessment emphasized multidisciplinary team approaches, which were important to identify a condition that was, at the time, considered both new and rare. A 2018 review of guidelines for diagnosis found consistent recommendations for multidisciplinary teams, but with little agreement on which team members or diagnostic tools were needed for accurate diagnosis (Penner, Anagnostou, Andoni, and Ungar 2018). Further, these recommendations were largely based on expert opinion, with scant empirical evidence informing clinical guidance. In their justification for continued use (or even requirement) of these models, some, including payors in the US, have identified the complex nature of differential diagnosis and individualized intervention planning as reasons that autism evaluation should be reserved for experts using a specific set of standardized tools (Kanne and Bishop 2021; National Collaborating Centre for Women's and Children's Health 2011).

Given the substantial resources required for comprehensive autism diagnostic evaluations, this rationale deserves careful interrogation. From a practical perspective, the temporal utility of developing a ‘comprehensive’ profile of a child's needs is limited, particularly in early childhood when developmental and behavioral changes occur rapidly. For instance, a survey of behavior therapists showed that while they considered information from a diagnostic evaluation, this information was often outdated by the time it reached them and they relied predominately on their own discipline‐specific assessment of the child to guide intervention planning (Caven et al. 2023). Although there is solid evidence that early behavioral intervention is effective for improving developmental outcomes in young children, science has not yet yielded precision‐focused differentiated intervention pathways, calling into question how comprehensive assessment data may contribute to individualized intervention recommendations. Further, co‐occurring conditions, which can have tremendous impacts on a child's support needs, may not yet be fully manifest and thus reliably differentiated or diagnosed in the early years (Forbes et al. 2023; Simonoff et al. 2008). Finally, the access implications of recommending that every child receives a ‘comprehensive’ evaluation must be considered. Unsurprisingly, longer assessment duration (required for more comprehensive evaluation) is associated with longer reported wait time for autism diagnostic evaluation (Penner, Anagnostou, and Ungar 2018). There will never be enough developmental and behavioral specialists to meet the needs of the growing population of autistic people (Bridgemohan et al. 2018; Weitzman et al. 2022). A change in perspective and approach is urgently needed.

## 2 Challenging History: Successful Innovation in Autism Diagnostic Evaluation

Application of a one‐size‐fits‐all approach to autism evaluation is a primary driver of delayed diagnosis and subsequent access to care. Research has clearly demonstrated that many young children with frank clinical presentations do not require specialist‐driven, resource‐intensive comprehensive evaluations for accurate identification of autism (de Marchena and Miller 2017; de Marchena et al. 2023; Thomas et al. 2024). This evidence, paired with the successful implementation of novel models of care that improve access to first‐time categorical diagnosis of autism, has paved the way for us to challenge our outdated approaches to care.

Building upon early foundational studies of primary care autism diagnosis (Swanson et al. 2014; Warren et al. 2009), our own teams (based in Ontario, Canada, and Indiana, USA) independently conducted and disseminated research demonstrating that community‐based pediatric clinicians can successfully apply streamlined assessment procedures and tools for high‐quality and accurate diagnosis of young children (McNally Keehn et al. 2023; Penner et al. 2023). Specifically, the evaluations in our studies involved nonspecialist administration of: (1) a brief interview to assess medical, developmental, and family history and the presence of key DSM‐5 focused autism features, and (2) an observational assessment, sometimes using a standard tool to quantify likelihood for autism. We have demonstrated that use of this simplified, efficient, community‐based approach to autism evaluation results in a high level of diagnostic accuracy, particularly when clinicians had high confidence regarding the presence of autism (i.e., positive predictive values ~90% in both studies). Both studies showed that there were almost no false negatives (i.e., over‐diagnosis of autism by community clinicians), but that community clinicians were not confident or did not have the level of expertise necessary to identify autism in some children with more complex profiles (i.e., those with higher developmental and adaptive functioning). Critically, in the Indiana study, the vast majority of those ‘false negative’ cases were flagged for referral to a specialist for second opinion expert evaluation (McNally Keehn et al. 2023), demonstrating the success of a tiered community‐specialist approach. Beyond diagnostic accuracy, our teams, and others have demonstrated that community‐based models of autism diagnosis are feasible, reduce diagnostic wait times, are acceptable to families, and have potential for streamlining connections to subsequent care and community support (McNally Keehn et al. 2023, 2020; Penner et al. 2023; Sohl et al. 2023; Hine et al. 2019; Harrison et al. 2017; Martin et al. 2024; Saunders et al. 2025). The innovative thinking behind these models provides a foundation for building out system strategies that similarly promote timely, local access to focused, right‐sized assessment across the life course.

## 3 A Personalized Approach to Autism Assessment Reflects Evolving Individualized Needs Across the Lifespan

Critics of streamlined categorical diagnostic assessment models argue that these models do not assess for all relevant co‐occurring conditions to build a holistic profile for an autistic person. We agree. Indeed, when autism is diagnosed in the early years, identification of most co‐occurring conditions and a detailed profile of strengths and challenges is not yet possible due to the developmental unfolding of most conditions. Adopting a personalized lifespan approach to autism assessment acknowledges the *ongoing* need to gather information about the autistic person's profile and to use this to help guide the right care and support at the right time. In this approach, the categorical diagnostic assessment is only one step on the autistic person's journey, albeit an important one. Decoupling the diagnostic evaluation from assessment of other domains of function, such as psychoeducational needs and mental health conditions, allows these to be completed when they are most reliable, valid, and informative (e.g., to inform contemporaneous learning modifications for a school‐aged child or needed interventions to support mental health of an adolescent). This approach recognizes the importance of assessing co‐occurring mental and physical health conditions during specific developmental touchpoints when these conditions are known to emerge (Lai et al. 2019), and acknowledges the relevance of different life transitions (Georgiades et al. 2022). It encourages each clinician to work to their full scope of practice, to engage existing community systems in collaborative assessment and care, and to refer to experts when the child and family need them, not simply because a specific assessor/assessment is required for service eligibility or because this is the historical *status quo*. This approach enhances opportunities for innovation, including implementation of technologies meant to support diagnostic accuracy and access (Klin 2025; Keehn et al. 2024). Perhaps most importantly, it recognizes that assessment needs of autistic people extend into adulthood, including into their elder years.

The personalized lifespan approach to autism assessment builds upon and extends national and international guidance. For example, autism diagnostic evaluation guidelines in both Australia (Whitehouse et al. 2018) and Canada (Brian et al. 2019) recognize that categorical autism diagnostic evaluation and related assessments identifying individualized support needs serve separate but complementary functions. *The Lancet Commission on the future of care and clinical research in autism* recommends a stepped‐care model of developmentally sequenced autism interventions (Lord et al. 2022). Our framework applies this stepped‐care approach to autism assessment, building upon the Commission's recommendations for diagnostic evaluation by explicitly considering what aspects of assessment could, and should, come *after* a categorical diagnosis is determined.

## 4 Implications for Clinical Practice

There are many clinical care implications from adopting a personalized lifespan approach to autism assessment. First, timely access to answer the most pressing assessment question(s) should receive priority over one‐time, or even repeated, ‘comprehensive’ approaches. Early categorical autism diagnosis enables access to supports that foster foundational developmental skills (Gabbay‐Dizdar et al. 2022) and should not be delayed only to access a subspecialist or to obtain additional information that does not directly impact immediate next steps. Given the prevalence of autism, all clinicians—general or specialist—need to recognize that they will encounter autistic people and consider what they are able to offer. Care encounters should be tailored to the needs of the autistic person and their family at that time. For instance, if a child is presenting for an autism evaluation and has a question of attention deficit/hyperactivity disorder (ADHD), both should be included in the assessment. Collateral sources of clinical information, such as school reports and therapy progress notes can also provide important information to update a person's profile of strengths and needs over time. Autistic people and their families benefit from having access to a ‘medical home’ where members of a medical team can synthesize and action this information and provide anticipatory guidance (Carbone et al. 2010). Finally, clinicians in the adult system, including those providing elder care, need to identify and/or develop ongoing assessment tools to best capture autistic quality of life and needs through the adult years.

## 5 Implications for Service Systems

A personalized lifespan approach to autism assessment allows us to decouple categorical diagnosis from other aspects of assessment such that various service systems and community resources can be engaged to meet the individual needs of the autistic person when and where they are most relevant. Take for example the two‐year‐old with frank autism presentation who receives a categorical diagnosis from their community primary care clinician and accesses behavioral interventions. Instead of receiving a cognitive assessment at the time of diagnosis, this child receives a psychoeducational assessment from their local school system to guide educational placement and planning during their school age years. As an adolescent, they receive an evaluation of vocational and adaptive skills to support the transition to adulthood. At each developmental touchpoint, the needs of this individual should first be addressed by the various health, educational, and vocational service systems embedded within their local community. Task shifting service provision from specialty care to local service systems (most notably applied in low‐ and middle‐income countries (Divan et al. 2021)) is a *simple but fundamental paradigm shift* that has the important potential of removing bottlenecks across service access points, freeing up specialists so that those who require their expertise can receive it without substantial delays. This shift challenges the notion that only specialists can care for autistic people and instead paves the way for accepting that expertise and resources can and should be distributed within communities.

To make this paradigm shift a reality, however, there is a lot of work to do—work that must be the shared responsibility of specialists, community services systems, and policy makers. Specialists must acknowledge that we cannot and should not be the sole owners of care for autistic people. Gatekeeping aligned with historical models stifles innovation and prevents us from using our expertise to build community capacity and design better systems. Instead, to move shared community‐specialty care of autistic people from an idea to a reality, professional roles and scope of practice must be clearly defined, there must be efficient methods for sharing information across clinicians and services systems, and payor models, reimbursement structures, and related disability entitlements must be aligned to support shared care across systems. Models such as ECHO Autism (Mazurek et al. 2017) and Indiana's Early Autism Evaluation Hub Network (McNally Keehn et al. 2020) are two examples among many programs that build and champion community‐based autism expertise.

In conclusion, a personalized lifespan approach to autism assessment is a clinical and system‐organization paradigm shift that values the ongoing need to collect and synthesize clinically relevant information about an autistic person across their life course. Categorical autism diagnosis is but one assessment event on this journey. To realize this vision, clinicians across the spectrum of care must be equipped and resourced to support their autistic clients, ensuring they can access relevant, timely assessments based on their current and evolving needs. Further, and perhaps most importantly, health systems and policies need to not only align with, but incentivize, innovative models of autism assessment that provide the right care and support at the right time.

## Conflicts of Interest

Dr. Penner reports paid consulting with Addis & Associates/Roche, the Province of Nova Scotia, and MedCounsel. She has received research funding from the Canadian Institutes of Health Research, Holland Bloorview Kids Rehabilitation Hospital Foundation, and Autism Speaks. Dr. McNally Keehn reports workshop/consultant fees from Purdue University, patent pending for an autism clinical decision support device, and research funding from Riley Children's Foundation, Indiana Department of Health, Spencer Foundation, Indiana Clinical and Translational Sciences Institute, and the National Institutes of Health.

## Data Availability

Data sharing not applicable to this article as no datasets were generated or analysed during the current study.
